# Neoatherosclerosis: A Distinctive Pathological Mechanism of Stent Failure

**DOI:** 10.31083/j.rcm2503095

**Published:** 2024-03-07

**Authors:** Mengting Jiang, Yu Zhang, Yan Han, Xiaohang Yuan, Lei Gao

**Affiliations:** ^1^Senior Department of Cardiology, Sixth Medical Center of Chinese PLA General Hospital, 100048 Beijing, China; ^2^Department of Clinical Training and Teaching, Tianjin University of Traditional Chinese Medicine, 301617 Tianjin, China

**Keywords:** neoatherosclerosis, in-stent restenosis, optical coherence tomography, very late stent thrombosis, atherosclerosis

## Abstract

With the development of drug-eluting stents, intimal re-endothelialisation is significantly inhibited by antiproliferative drugs, and stent restenosis transforms from smooth muscle cell proliferation to neoatherosclerosis (NA). As a result of the development of intravascular imaging technology, the incidence and characteristics of NA can be explored *in vivo*, with some progress made in illustrating the mechanisms of NA. Experimental studies have shed light on the molecular characteristics of NA. More critically, sufficient evidence proves NA as a significant cause of late stent failure. Treatments for NA are still being explored. In this review, we summarise the histopathological characteristics of different types of stent NA, explore the potential relationship of NA with native atherosclerosis and discuss the clinical significance of NA in late stent failure and the promising present and future prevention and treatment strategies.

## 1. Introduction

Percutaneous coronary intervention (PCI) is the first choice of treatment for coronary heart disease. However, the incidence of in-stent restenosis (ISR) remains up to 10% [1]. In-stent neoatherosclerosis (ISNA) is characterised by the accumulation of foamy macrophages, necrotic core formation and calcification of intima at the site of stent implantation (in-stent or within 5 mm of stent edge) and is considered an essential cause of ISR [2]. Drug-eluting stents (DES), which were later identified as first-generation DES (G1-DES), have gradually been selected to replace bare metal stents (BMS) for modifying prognosis, with ISNA remaining a concerning problem to be solved. In G2-DES, clinical results showed improvement in the complications of late and very late thromboses, which were reduced by the optimisation of stent materials, strut volume and polymer sustained-release system; however, these developments are still insufficient to avoid the development of neoatherosclerosis (NA) [3]. *In vivo* intravascular imaging, which involves intravascular ultrasound (IVUS) and optical coherence tomography (OCT), exhibits significant advantages in the diagnosis and treatment during PCI, especially of ISNA. In comparison with IVUS, OCT is specialised to function in high resolution, which enables the full display of the NA in and around stents. Herein, we review the histopathological characteristics of different types of stent NA, the potential relationship of NA with native atherosclerosis (AS), the clinical significance of NA in late stent failure and the current and future promising prevention and treatment strategies.

## 2. Differences between Pathology and Intravascular Imaging of NA

NA is histologically described as the accumulation of lipid-laden foamy macrophages in the neointima, with or without necrotic core formation or calcification [4]. The early manifestation of NA involves the aggregation of foamy macrophages around the strut of the stent or on the lumen surface, with the necrotic core composed of discrete cell-free fragments, rich free cholesterol and extracellular matrix. Intraplaque haemorrhage can be observed accompanied by fibrin deposition, which is possibly derived from the lumen surface through cracks or ‘leaks’ from the adventitial vasa vasorum. This early feature then induces the development of fibroatheromatous plaque. Thin-cap fibrous AS (TCFA) may result in the rupture of the plaque, which potentially develops into clinically adverse coronary events. Microhemorrhage in the peristrut region of stents, which could be caused by the different compliance of the rigid stent and relatively softer artery wall, can also be observed [5]. Calcification is another feature of NA, and it includes microcalcification and calcified sheets, where the former may originate from the apoptosis of foamy macrophages or smooth muscle cells [6] and the latter from collagen, extracellular matrix and smooth muscle cells; both apoptosis types usually occur after long-term implantation, especially in BMS-related NA. In DES-related NA, fibrin deposited in the peristrut regions is more commonly reported [7].

Pathology results are considered the gold standard in NA diagnosis. However, pathological specimens are difficult to obtain. *In vivo* intravascular imaging, such as IVUS and OCT have played an increasing role in clinical diagnosis and treatment. Compared to IVUS, OCT can attain higher resolution, and therefore has the capacity to comprehensively display neoatherosclerosis within stent segments to contribute to evaluating the morphological features of NA [8].

ISNA, as defined by OCT, refers to the presence of lipid-containing neointima or calcification in culprit stents with longitudinal extension ≥1 mm [9, 10]. Neointima lipid is characterised as a diffusely bordered, signal-poor region with rapid signal attenuation and covered with rich signal fibre caps [11]. Calcified neointima is characterized by well-defined areas with poor signal and clear boundaries [12]. Macrophages show high reflection and strong attenuation in dot or stripe structures, with radial light and shadow. Angiogenesis is displayed as holes or tubular structures with signal differences of diameters ≥50 and ≤300 mm, which appear in at least three consecutive frames [13] (Table 1).

**Table 1.** **Difference between pathology and OCT in NA detection**.

Index	Pathology	OCT
Lipid core	+++	++ (the thickness cannot be measured)
Plaque property	+++	++
Macrophage infiltration	+++	++
Fibrous cap thickness	+++	+++ (can be accurately measured)
Microcalcification	+++	+
Flake calcification	+++	++
Massive haemorrhage	+++	+
Microhaemorrhages	+++	-
Microvessels	+++	++

OCT, optical coherence tomography; NA, neoatherosclerosis.

There are limitations to diagnosing NA with OCT. The limited resolution of OCT can cause microhemorrhage and microcalcifications that could be observed on histology to be missed when utilizing OCT alone [14]. Due to the limited penetrance of OCT, it can be difficult to adequately estimate the overall lipid burden of a necrotic core. However, surrogate measurements such as lipid angle have been validated to better estimate lipid plaque area [15]. Adventitia is also difficult to observe due to the insufficient penetration and obstruction by the struts from implanted stents [16]. It is also difficult to determine the boundary of calcification which is near the adventitia. There is an incomplete agreement between OCT and histopathologic analysis of blood vessels in determining the presence of NA. A study investigating the accuracy of the characterization of atherosclerosis by OCT compared to histopathology found that OCT demonstrated a sensitivity and specificity ranging from 71% to 79% and 97% to 98% for fibrous plaques, 95% to 96% and 97% for fibrocalcific plaques, and 90% to 94% and 90% to 92% for lipid-rich plaques [17]. A possible explanation for the overestimation of NA by OCT is that fibrin accumulation, granulation tissue, and highly organized thrombus can all share a similarly low intensity signal as a necrotic core. Therefore, the limitations of OCT imaging need to be considered when interpreting clinical data. OCT automatic quantification of signal attenuation can perform sensitive identification of foam cells in the intima after stent implantation, which is in robust agreement with the pathological verification. This is a method developed to improve the accuracy of NA diagnosis by OCT [16]. Several types of double-probe catheters have also been gradually applied, such as combined near infrared spectroscopy (NIRS)-IVUS, IVUS-OCT, OCT-NIRS, OCT-near infrared fluorescence (NIRF) molecular imaging, IVUS-NIRF, IVUS intravascular photoacoustic imaging and combined fluorescence lifetime-IVUS imaging [18, 19]. The introduction of new imaging technology is expected to modify the accuracy of diagnosis of NA *in vivo* (Table 2, Ref. [4, 20, 21, 22, 23, 24, 25, 26, 27, 28, 29, 30, 31, 32, 33, 34, 35]).

**Table 2.** **Results of pathological and OCT studies on the evaluation of the incidence of NA**.

Author	Year	Subjects	Type of stent	Observation type	Time after PCI	NA incidence
Takano *et al*. [20]	2009	Patients with OCT follow up	BMS, n = 21	OCT	5 yr	67%
Kang *et al*. [21]	2011	Patients with intimal hyperplasia >50% of stent area	DES, n = 50	OCT	32.2 mon	90%
Nakazawa *et al*. [4]	2011	Autopsy cases after PCI	BMS, n = 142	pathology	2160 d	16%
			DES, n = 157		420 d	31%
Kim *et al*. [22]	2012	Patients with OCT follow up	DES, n = 76	OCT	9 mon	15%
					2 yr	28%
Yonetsu *et al*. [23]	2012	Patients with neointimal thickness >100 um	BMS, n = 73	OCT	Mean 26.9 mon	47%
			DES, n = 106			
Lee *et al*. [24]	2013	Patients with >50% CSA neointimal stenotic lesions	BMS, n = 24	OCT	70.7 mon	35.50%
			DES, n = 128			
Otsuka [25]	2014	Autopsy cases after PCI	SES, n = 73	pathology	270 d	35%
			PES, n = 85		210 d	19%
			EES, n = 46		200 d	29%
Lee *et al*. [26]	2015	Patients with >50% neointimal CSA stenosis	G1-DES, n = 101	OCT	55 mon	46%
			G2-DES, n = 111		12 mon	11%
Kuroda *et al*. [27]	2016	Patients with OCT follow up	BMS, n = 37	OCT	>1 yr	17%
			DES, n = 277			
Jinnouchi *et al*. [28]	2017	Patients with ISR after PCI	G2-DES, n = 324	OCT	212 d	2.82%
					632 d	15.70%
Tomaniak *et al*. [29]	2018	Patients with OCT follow up	DES, n = 39	OCT	3 yr	23.10%
					9 yr	30.80%
Kobayashi *et al*. [30]	2018	Patients with ISR after PCI	G1-DES, n = 102	OCT	55 mon	27.20%
			G2-DES, n = 114		32 mon	32.40%
Hoshino *et al*. [31]	2019	Patients with OCT at >3 years after PCI	BMS, n = 25	OCT	5.1 yr	25.70%
			DES, n = 88			
Sumino *et al*. [32]	2021	Patients with OCT performed between 3 and 7 years after PCI	BMS, n = 72	OCT	4.8 yr	19.30%
			DES, n = 236			
Nakamura *et al*. [33]	2021	Patients with ISR after PCI	BMS, n = 64	OCT	732 d	47%
			DES, n = 241			
Chen *et al*. [34]	2022	Patients with ISR after PCI	G2-DES, n = 512	OCT	2.8 yr	28.50%
Yuan *et al*. [35]	2023	Patients with ISR after PCI	Male, n = 188	OCT	6.2 yr	82%
			Female, n = 42		4.4 yr	62.80%

OCT, optical coherence tomography; NA, neoatherosclerosis; BMS, bare metal stent; DES, drug-eluting stent; G1-DES, first-generation DES; G2-DES, second-generation DES; PCI, percutaneous coronary intervention; SES, sirolimus-eluting stent; PES, paclitaxel-eluting stent; EES, everolimus-eluting stent; CSA, cross-sectional area; ISR, in-stent restenosis.

## 3. Relationship between NA and AS

Native AS is a condition characterised by lipid accumulation, fibrous tissue hyperplasia and calcium deposition in the intima and is accompanied by the gradual degeneration and calcification of the arterial middle layer. Native AS refers to primary AS, while NA refers specifically to AS occurring within the stent. NA clusters at native plaques. It implies a relationship between native plaques and NA. Following PCI with stent placement, the degree of residual plaque burden at the time of stent implantation has been found to be correlated with increased risk for ISR [36]. Various studies have demonstrated the relationship between native plaque burden and ISNA [31, 37]. Kang S *et al*. [38] considered plaque burden around the stent a predictor of intimal hyperplasia within 6 months to 2 years. Andreou I *et al*. [39] also reported a significant relationship between the reduction of plaque area after stent implantation and the development of NA at follow-up. These findings may suggest a connection between NA and native AS.

The potential mechanisms of native plaque-affecting NA formation after stent implantation are summarised as follows. (1) Inflammation and chemokines in native atherosclerotic plaque may contribute to the gathering of inflammatory cells and growth factors, which elicits the aggregation of local inflammatory factors that induce a sustained inflammatory reaction and promote the formation of NA. (2) For unstable plaques, the stent can embed in the large necrotic core which in turn can inhibit release of the drug from DES causing endothelial dysfunction and delayed healing.

Studies have also proposed a relationship between NA formation and the degree of underlying AS in non-target lesions. An OCT analysis involving 88 patients indicated that 5 years after stent implantation, the presence of NA was correlated with the progression of native atherosclerosis in non-target lesions and that the need for non-target lesion revascularization was correlated with NA in the target lesion [40]. Another study, with a 3-year follow-up noted an association of NA formation after G1-DES implantation with AS progression in non-stented segments [41]. Consistent with these results, Xing *et al*. [42] reported that plaque characteristics, such as minimum lumen diameter and plaque with lipid core length, are closely related to ISNA formation.

Endothelial shear stress has been related to plaque formation in native coronary vessels, thus establishing the importance of the local hemodynamic environment in AS development and progression. Therefore, the changes brought upon by stent deployment could have similar effects in adjacent native AS progression [43]. These mechanisms need to be investigated further.

In addition, a study involving 212 patients demonstrates the presence of a positive correlation between the degree of neointimal hyperplasia after stent implantation and the presence of NA. This association is independent of stent type and time from implantation and suggests a possible pathogenic link between the two processes [44].

## 4. Morphometric Features of NA in Different Stent Types

The types of stents have been gradually innovated from BMS, G1-DES and G2-DES to bioresorbable vascular scaffolds (BVS), where BMS served widely as the first stent type in clinical PCI treatment. Native AS development takes years to decades, while ISNA can form in months to years [45, 46]. The capacity of carrying antiproliferative drugs was later developed in G1-DES to inhibit the proliferation of NA, which involves a sirolimus-eluting stent (SES) and paclitaxel-eluting stent (PES). However, given the immune response induced by G1-DES to stent polymers and endothelial dysfunction caused by antiproliferative drugs, NA occurs earlier than with BMS and presents a higher morbidity [4].

G2-DES, which includes a everolimus-eluting stent (EES), a zotarolimus-eluting stent and a biolimus-eluting stent, which are made of cobalt-chromium alloy instead of stainless steel in order to obtain optimal flexibility and conformability and promising biocompatibility. The application of G2-DES greatly modified the clinical outcomes and reduced the complications of late and very late thromboses; however, they still failed to inhibit the development of NA [47]. BVS was then introduced to clinical practice, however, ISNA was still reported in BVS in the middle and late stages [48]. Here we analyse the morphological diversity of ISNA caused by different types of stents both by pathology and OCT.

### 4.1 Autopsy

Autopsy results showed the main component of NA after BMS implantation is the extracellular matrix, including proteoglycan, hyaluronic acid and type III collagen, with a high proportion of smooth muscle cells. Three to four months following stent implantation, type 1 collagen increased and the extracellular matrix decreased, which seems to slow the progression of endothelial coverage of BMS. The neointima gradually stabilised after 18 months [4, 49].

The early neointima after DES implantation is thinner than that after BMS implantation, and is mainly composed of peristrut fibrin. Neointima after DES has minimal vascular smooth muscle cells, proteoglycan-rich extracellular matrix and poorly covering endothelial cells [45]. The potentially protective upregulation of calcium-regulating proteins was noted in the early neointima from DES compared to the neointima of BMS [50]. Over time, the neointimal components of restenotic DES exhibit increased proteoglycan deposition and fewer smooth muscle cells in comparison with BMS [51]. A study examining 299 autopsies and 406 lesions reported that the earliest foam macrophage accumulation was 70 days after PES, 120 days after SES but as long as 900 days after BMS. A necrotic core was observed at 270 days after PES and 360 days after SES. The unstable characteristics of NA, namely, TCFA and plaque rupture in the stent, were found within 2 years after the implant of G1-DES and 5 years after BMS [4]. The differing results between BMS and DES can be related to the capability of antiproliferative drugs to inhibit the proliferation, migration, and survival of endothelial cells, thereby allowing lipid-laden foamy macrophages to ‘leak’ into the stented arteries and thus accelerating the development of NA. This condition also differed from native coronary AS that have been occurring over the decades.

G2-DES-related NA, especially for EES, exhibit less inflammation, more complete neointimal coverage and re-endothelialisation. Otsuka *et al*. [25] reported that the earliest period of NA in EES was 270 days, which was longer than that in SES (120 days) or PES (70 days). No TCFA nor plaque rupture was observed in EES. No EES showed a hypersensitivity reaction, while 8% of SES showed a hypersensitivity reaction. However, there was no significant difference in the incidence of NA (42% in EES vs. 60% in SES vs. 27% in PES) [25].

To minimise the downsides of life-long mechanical and biological stresses induced by permanent implantation, scientists introduced BVS to clinical practice because absorbable materials can still permit drug delivery and provide transient vessel support after PCI by avoiding retraction and acute occlusion. In several months or years, BVS materials will be completely bioabsorbed, and the structure and systolic/diastolic functions of the coronary artery will be regained. Multiple randomized controlled trials have compared outcomes between BVS and DES. BVS and DES reported similar results in 1 year [52, 53, 54, 55], the risk of myocardial infarction after BVS was higher than DES with a median follow-up of about 2 years [56]; the risk of stent thrombosis after BVS significantly increased after 3 years compared with DES [57], the incidences of thrombosis and developed NA was higher in the BVS group than those in the DES group after 5 years of follow-up [58, 59]. These results indicate that the safety of BVS remains a concern. To date, autopsy pathological studies of BVS are rare. Van Ditzhuijzen *et al*. [60] reported the pathology after 6 months of BVS implantation in a pig model, revealing that the NA was heterogeneous, lipid-laden and rich in calcification with incomplete intima coverage; this finding indicated NA as the main cause of the failure of BVS in the long-term.

### 4.2 Autopsy

As determined by OCT, the prevalence of NA increases with time [61]. Habara *et al*. [62] compared the features of early (≤1 year) and late restenosis (>5 years) after BMS, revealing the more frequent observation of heterogeneous intima in late ISR than those in the early stage.

Nagoshi *et al*. [3] analysed the characteristics of NA after DES and BMS implantation using qualitative and quantitative OCT and observed that the NA lesion of G1-DES was mainly the layered type and composed of collagen fibres and smooth muscle cells, and the BMS homogeneously consisted of proteoglycan, cell matrix and organised thrombus. Yamaguchi *et al*. [63] compared OCT findings between two groups with ISR—which they defined as the ‘jump-up’ or ‘gradual progression’ groups and found that the ‘jump-up’ group more commonly had heterogenous OCT morphology while the layered or homogenous pattern was more commonly seen in the ‘gradual progression’ group. As the time after DES implantation increased, there was an increased incidence of TCFA and microvessels [64]. Microvessels serve as the transmission route for inflammatory cells and red blood cells during lipid plaque formation, which indicates the evolution from stable to unstable plaque [65]. Hada *et al*. [66] reported a 10-year follow-up after BMS or G1-DES and G1-DES showed more frequent uncovered and malposed struts within stents, which has in turn been found to be associated with an increased risk for very late stent thrombosis (VLST).

Another cohort study comparing BMS, G1-DES and G2-DES using OCT showed a higher rate of detection of NA in G1-DES, although a thinner fibrous cap in BMS as well as a greater extent of lipid extension in BMS [67]. Kobayashi *et al*. [30] indicated that although the rate of NA detection in G1-DES and G2-DES was similar, there were significant differences in NA characteristics between G1-DES and G2-DES. Compared with G2-DES, NA in G1-DES has increased lipid length, larger lipid arch, prevalence of a 360-degree lipid arc and thinner fibrous cap [30]. This result indicates that the stability of NA may be better in G2-DES.

After the implantation of BVS, NA formation begins earlier and continues to develop. Moriyama *et al*. [68] reported that in the inner part of the stent, the incidence of NA was almost 100% after 5 years. Calcification, neovascularisation, macrophage infiltration and lipid plaques generally occurred in the inner and outer parts of the stent. Compared with BMS or DES, the inflammation elicited by proteoglycan after scaffold resorption remains a potential reason for the accelerated formation of NA [68, 69] (Table 3).

**Table 3.** **Morphological differences in NA among different kinds of stents**.

Index	Pathology			
	BMS	G1-DES	G2-DES	BVS
Endothelial cell coverage	Good	Poor	Better	Poor
Smooth muscle cell	Visible	Rare	Rare	-
Inflammatory reaction	More	More	Less	-
Calcification	Rare	Rare	Rare	Common
Plaque property	-	-	-	Heterogeneity
Time of foam macrophage aggregation	900 d	70–120 d	270 d	-
Necrotic core formation	900 d	270–360 d	-	-
TCFA and in-stent plaque rupture	5 yr	2 yr	Not observed	-
Index	OCT			
	BMS	G1-DES	G2-DES	BVS
Plaque property	Homogeneous is common	Layered is common	-	-
Minimum fibre cap thickness	Thinnest	Thick	Thickest	-
Longitudinal length	Longest	Shorter	Shortest	-
Calcification	No significant difference			Common
TCFA	No significant difference			-
Microvessels	No significant difference			Common

NA, neoatherosclerosis; BMS, bare metal stent; G1-DES, first-generation DES; G2-DES, second-generation DES; BVS, biodegradable stent; OCT, optical coherence tomography; TCFA, thin-cap fibrous atherosclerosis.

## 5. Potential Mechanisms of NA Formation and Development

**Fig. 1.**
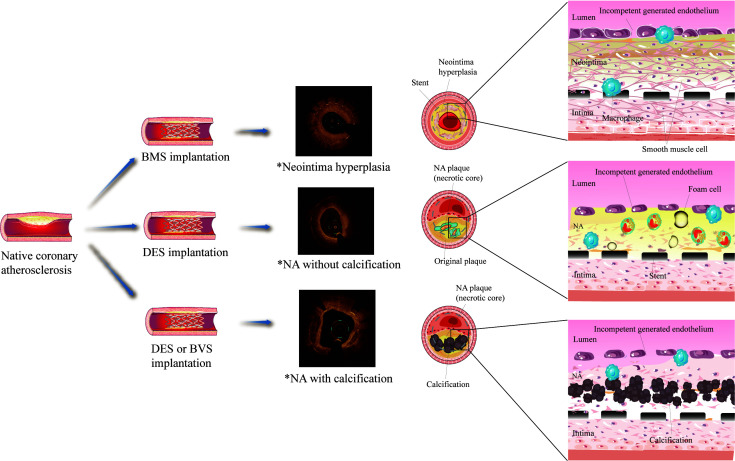
**Schematic of the mechanism of neoatherosclerosis (NA)**. *OCT images were collected from clinical cases. NA, neoatherosclerosis; BMS, bare metal stent; DES, drug-eluting stent; BVS, biodegradable stents.

Many etiologies for NA formation and development have been proposed. In the following sections, we summarize the findings supporting three unique etiologies, endothelial dysfunction, inflammation, and hemodynamic changes (Fig. 1).

### 5.1 Autopsy

The incidence of NA is higher with DES than with BMS [4]. It has been proposed that the incomplete re-endothelialization and endothelial dysfunction induced by antiproliferative agents, such as sirolimus and paclitaxel, can be responsible for this observed difference. Jabs *et al*. [70] demonstrated that sirolimus exposure led to both endothelial-dependent and endothelial-independent vasorelaxation impairment. They further demonstrated that sirolimus exposure led to an increase in free radical production, both from cytosolic nicotinamide adenine dinucleotide phosphate as well as from the mitochondrial respiratory chain. Reactive oxygen species play an important role in caveolae formation by upregulating and activating caveolin-1, a primary structural protein of caveolae, therefore increasing lipid uptake and retention in endothelial cells and causing endothelial dysfunction which could lead to atherogenesis [71, 72, 73].

Vascular endothelial (VE)-cadherin, which maintains cell–cell integrity, is also affected by antiproliferative drugs as reactive oxygen species accumulate. The impaired intercellular junctions allow the entrance of lipoproteins into the subendothelial space, which can initiate NA formation. Several studies have examined VE-cadherin expression after stent implantation by comparing the biodegradable polymer DES (BP-DES) with durable polymers DES (DP-DES) and BMS and found decreased VE-cadherin expression of DES compared to BMS, which was also associated with different endothelial histology. However, BP-DES showed less suppression of VE-cadherin relative to DP-DES [74, 75].

Recent research indicated that smooth muscle cell-derived CXC chemokine ligand-10 prevents endothelial healing through phosphoinositide 3-kinase γ-dependent T cell response, which may provide new strategies for NA treatment [76].

### 5.2 Inflammation

Inflammatory reactions also play a crucial role in the formation of NA and are considered a robust predictor of essential complications of stent implantation, such as ISR and late stent thrombosis. In the initial phase after stent implantation, an acute inflammatory reaction occurs as a consequence of the arterial injury. Balloon expansion and stent implantation cause medial injury during the procedure, along with tissue factor release [51]. The expression of adhesion molecules (such as intercellular adhesion molecule-1 and vascular cell adhesion molecule-1) promotes the recruitment of inflammatory cells (monocytes, T cells and neutrophils). Chemokines (monocyte chemoattractant protein-1 or interleukin (IL)-8) and growth factors are produced by endothelial and smooth muscle cells. Inflammatory factors are released through the activation of cytokines, developing into a local ‘inflammatory factor storm’ [25, 77]. Influenced by stent implantation and drug release, intimal hyperplasia is inhibited, accompanied by the delayed healing of the injured part, thus inducing a sustained inflammatory reaction. In the following weeks after stent implantation, a chronic inflammatory process may occur. Chronic production of cytokines and growth factors causes phenotypic changes of smooth muscle cells and their migration into the intima [78]. In the late phase, over the months after stent implantation, smooth muscle cells shift towards greater extracellular matrix synthesis, rather than a proliferative activity, thus forming a neointima rich in extracellular matrix [79]. Promoted by chemokines, monocytes migrate to the endothelium and transform into macrophages, thereby forming a necrotic core that acts as the main component of ISNA. The infiltration of foamy macrophages gradually forms a TCFA, which conceivably increases the risk of plaque formation [80, 81, 82].

In addition, an individual’s allergic inflammatory response to stent implantation is an important factor. The metal stent struts and the polymer may promote local recruitment and activation of effector cells of allergic inflammation [79]. In an experiment comparing the histopathological features of restenosis tissue after balloon angioplasty and stent implantation, eosinophilic infiltration is present in ISR tissue of bare-metal stent-treated patients, but rarely in postballoon restenosis tissue [83]. This suggests that polymer or metal may be the cause of allergic inflammation. In addition, compared with BMS, DES are more likely to be observed with eosinophilic infiltration [84]. Animal experiments showed that polymers can produce hypersensitivity reactions when implanted in swine coronary arteries [85]. A study by Byrne *et al*. [86] showed that the permanent polymer DES had more significant late lumen loss after 6–8 months than the non-polymer DES. These suggest that polymer-induced inflammation plays a key role in DES restenosis. Allergic inflammation leads to delayed arterial healing, incomplete stent re-endothelialization, and stent malapposition, which may lead to ISNA formation [87].

### 5.3 Haemodynamic Disorder

Stent-induced flow disturbances are another factor affecting the formation of ISNA. After stent implantation, the non-streamlined stent strut intervenes with the blood flow conditions at the proximal and distal ends of the luminal surfaces of the stent (‘candy-wrapper effect’) [88, 89], which can induce a phenotypic change of endothelial cells as well as increase transmembrane protein expression. The expression of connexin intercellular adhesion molecule-1 and vascular cell adhesion molecule-1 is upregulated in the peristrut regions, thereby promoting the adhesion and migration of monocytes into the intima and their transformation into foamy macrophages, which later form necrotic cores [90, 91]. Early thrombosis after stent implantation is mainly described as a process of arterial healing with fibrin and platelet aggregation, and the continuous release of stent-coated drugs and local haemodynamic changes inhibits fibrin degradation, resulting in the continuous existence of thrombus material at the stent site [92, 93].

## 6. NA as the Main Mechanism of Late Restenosis and Stent Thrombosis

### 6.1 NA and ISR

ISR is generally defined as the stenosis of the coronary artery segment or lumen with a decrease of the diameter by at least 50% within 5 mm of the stent edge [94]. The mechanisms underlying ISR are multifactorial and include both mechanical (e.g., insufficient stent expansion or stent fracture) and biological etiologies. A study on 171 cases of G2-DES restenosis demonstrated that intimal hyperplasia, which resulted from incomplete stent expansion, was the primary cause of ISR in one-third of patients that developed early ISR (<1 year) and two-thirds of those that developed late ISR (>1 year), although 28.9% of patients with late ISR also demonstrated NA [95]. In another study, NA was detected in 37% of early ISR lesions in 185 patients with OCT, with a median follow-up of 180 days [96]. These studies suggest a possible etiologic role of NA in the development of both early and late ISR, although there is a higher incidence of NA in cases of late ISR.

The impact of NA on the prognosis after PCI for ISR is still controversial. An observational study included 64 patients with BMS and 241 patients with DES, of which 47.0% (147 lesions) showed NA. The results of multiple regression analysis indicated that NA acted as an independent predictor of clinically driven target lesion revascularisation [33]. Another study including 64 patients with ISR found 36% had developed NA as determined by OCT. It seems that the occurrence of NA may be related to the prognosis of patients with ISR after PCI. However, among the patients with or without NA, the angiographic follow-up of 6–9 months reported no difference in restenosis (24% vs. 15%; *p* = 0.49). During the 3-year follow-up, the incidence of major cardiovascular adverse events showed no significant difference (13% vs. 12%; *p* = 0.93). These results may suggest that the NA defined by OCT does not affect the acute and long-term prognosis of ISR patients after DES treatment [97]. More evidence from clinical trials is required to determine the impact of NA on ISR outcome prediction.

A comparison of the development of NA depending on stent type was also performed. Song *et al*. [98] evaluated ISR lesions as determined by OCT and identified an overall incidence of NA of 38.7% in all stent types (53.8% BMS, 65.1% G1-DES, 23.0% G2-DES). Among G2-DES, stent under expansion, fracture and deformation were more frequently detected, and thrombosis was more commonly found in G1-DES [98]. In another study, 212 ISR patients treated with DES were investigated, and NA was confirmed in 27.4% of the lesions by OCT. The incidence of NA was lower in the G2-DES group (10.8% vs. 45.5%; *p* < 0.001), and the stent age was shorter than that in the G1-DES group (12.4 months vs. 55.4 months; *p* < 0.001). After adjusting for cardiovascular risk factors, DES types (G1-DES and G2-DES) were not reported as independent predictors of NA [24]. In general, some modifications were made in the material, structure and coating of the G2-DES to reduce the incidence of NA. However, they still failed to solve complications, such as fractures and deformation of stents.

### 6.2 NA and VLST

VLST refers to stent arterial thrombosis that occurs more than 1 year after implantation. Despite its rare prevalence, VLST remains a highly studied entity due to the high morbidity and mortality associated this condition. Taniwaki *et al*. [2] reported the OCT results of 64 patients with VLST after DES, with the findings revealing NA as the potential mechanism in 27.6% of cases. An OCT examination of 134 VLST patients indicated that in-stent plaque rupture was the most common cause of VLST (31%), with a median duration of 5.95 years after any stent implantation, accounting for 69% in the NA group [99]. A multicentre study of 98 patients from South Korea also suggested NA as the most common cause of VLST (34.7%), followed by malposition (33.7%) and uncovered struts (24.5%) [100]. These results confirmed that the rupture of NA plaque after stent implantation is a critical cause of VLST. Late NA that has unstable histological features, such as a large necrotic core and thin fibrous cap, can contribute to the rupture of plaque in the stent and cause VLST [47, 101].

Very-late scaffold thrombosis (VLScT) after BVS is different from VLST after DES due to the use of bioresorbable materials. Yamaji *et al*. [102] reported that scaffold discontinuity (an absorption-related phenomenon not encountered by metal stents) was the most common underlying mechanism of VLScT (42.1%), followed by malapposition (18.4%) and NA (18.4%), at a median of 20 months follow-up. After BVS, VLScT occurs earlier than BMS, and it possibly results from the scaffold discontinuity via a unique resorption-related process.

## 7. Progress in the Treatment and Prevention of NA

### 7.1 Systemic Therapy

#### 7.1.1 Smoking Cessation

**Fig. 2.**
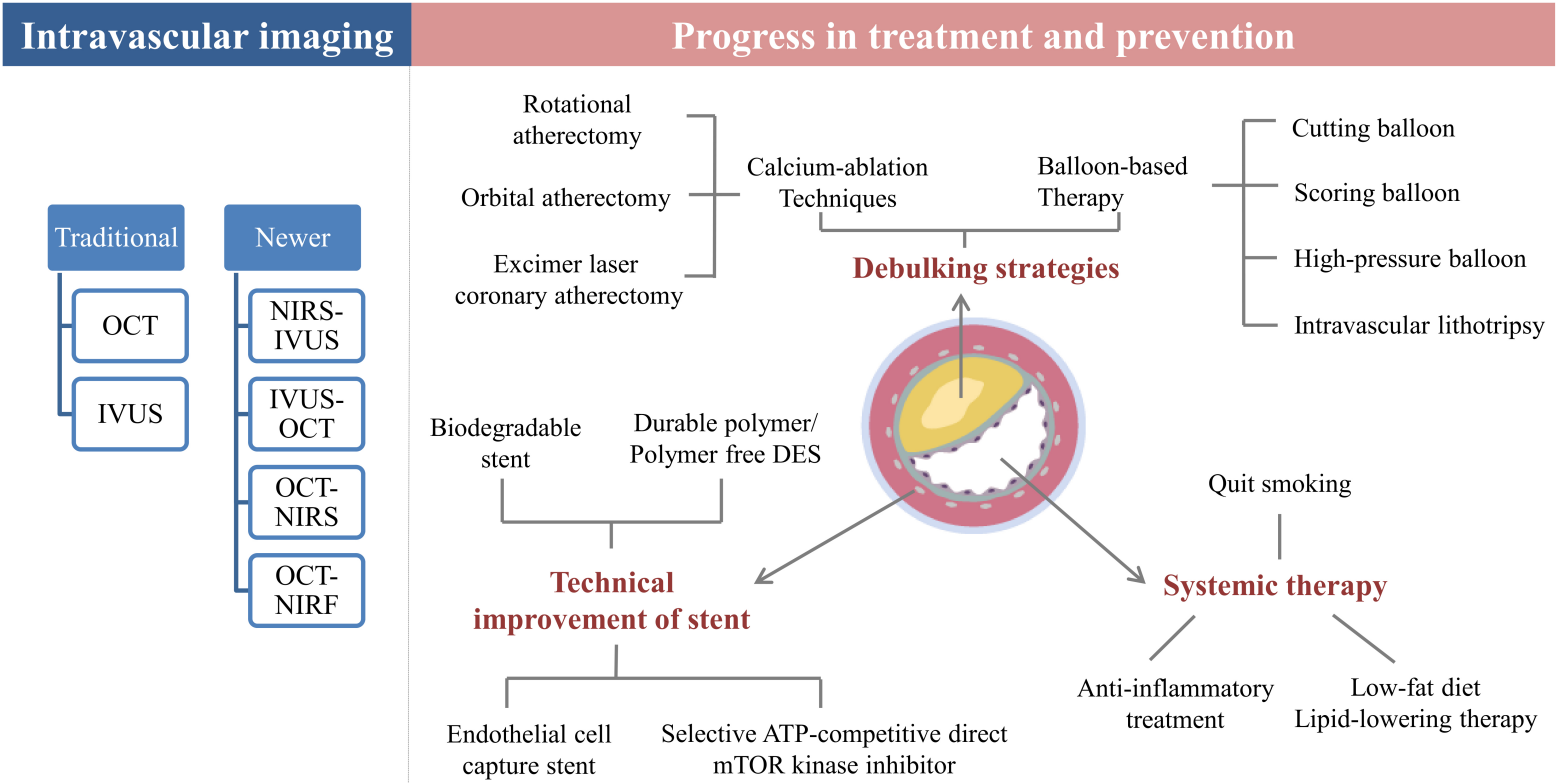
**Progress in the treatment and prevention of neoatherosclerosis (NA)**. OCT, optical coherence tomography; IVUS, intravascular ultrasound; NIRS, near-infrared spectroscopy; NIRF, near-infrared fluorescence; mTOR, mammalian target of rapamycin; DES, drug-eluting stent; ATP, adenosine triphosphate.

No agreement has been met in regard to the influence of smoking on NA. Gao *et al*. [103] reported a higher incidence of uncovered struts in nonsmokers in comparison with current smokers (13.3% ± 13.3% vs. 6.7% ± 8.3%; *p* = 0.001), with a more heterogeneous pattern of neointima shown in current smokers. Yonetsu *et al*. [104] compared the characteristics of patients with or without NA and reported current smoking habits as an independent predictor of NA after stenting. Nicotine stimulates the proliferation, migration and angiogenesis of bovine pulmonary artery endothelial cells *in vitro*, which may be promoted by improving strut coverage [105]. On the one hand, cigarette smoke promotes the expression of oxidants and carbon monoxide, which may lead to endothelial injury and AS [106]. The effect of smoking on NA requires further investigation (Fig. 2).

#### 7.1.2 Low-Fat Diet and Lipid-Lowering Therapy

Hypertriglyceridemia is considered an independent risk factor of ISR after PCI [107]; non-fasting hypertriglyceridemia is a residual risk factor after statin therapy, and lipoproteins rich in triglyceride are considered to result in intimal cholesterol deposition, NA, pro-inflammation and apoptosis [108]. Serum low-density lipoprotein cholesterol concentrations have received more attention in NA. Low-density lipoprotein cholesterol is an independent predictor of NA incidence [109]. With the introduction of lipid-lowering therapy to reduce the low-density lipoprotein cholesterol level, statins have been shown to prevent non-homogeneous variations in the neointima and to increase the cross-sectional area of the neointima [110]. However, whether the intervention using diet or drugs can reduce the risk of ISR in patients with coronary heart disease after direct PCI remains to be determined.

#### 7.1.3 Anti-Inflammatory Treatment

The efficacy of anti-inflammatory therapy for NA has been validated. A study of a rabbit model reported that intravenous methotrexate treatment can stabilise NA for DES while lowering the levels of pro-inflammatory cytokines (serum IL, adhesion molecule and nuclear factor-κB p65) and elevating the level of an anti-inflammatory cytokine (IL-10) [82]. Pei *et al*. [111] demonstrated that berberine (a plant extract used in traditional Chinese medicine) did inhibit nuclear factor-κB signalling, but activates AMPK signalling to exert its inhibitory effects on macrophage activation, which is expected to reduce restenosis and NA formation after stenting. Chen *et al*. [81] observed that curcumin may attenuate the inflammation and ISNA caused by poly-l-lactic acid degradation via peroxisome proliferator-activated receptor γ signal pathway *in vitro*; however, the related *in vivo* experiments are lacking. Targeting pro-inflammatory pathways can start a new era in the prevention of NA.

### 7.2 Systemic Therapy

Technical modifications of the stent include BVS, polymer improvement, direct mammalian target of rapamycin (mTOR) kinase inhibitor DES and endothelial cell capture stent, of which BVS has been discussed above.

#### 7.2.1 BP-DES and Polymer-Free DES

Biodegradable polymer-DES and polymer-free DES have been hypothesized to slow the progression of NA through modifying incomplete endothelialization and hypersensitivity reactions that can be characteristic of durable-polymer DES. A study involving 90 patients followed up for 18 months showed similar incidences of NA between BP-DES and DP-DES (11.6% vs. 15.9%; *p* = 0.56) [112]. OCT analysis of 311 patients and 319 lesions during the median follow-up of 4 years demonstrated a lower incidence of NA in the BP-DES group in comparison with the DP-DES group (5.2% vs. 14.5%; *p* = 0.008) [11]. A possible explanation is that BP-DES provides a better environment for endothelial healing as the polymer gradually degrades. A multicentre, prospective, observational study of 105 patients discovered better stent coverage and plaque stability of polymer-free DES at 12 months in comparison with DP-DES (*p* < 0.001) [113]. Compared with BP-DES, polymer-free DES exhibited more complete strut coverage (*p* < 0.001) [114], which is consistent with improved endothelial healing; however, an extended follow-up of polymer-free DES as a newly introduced therapy is still required.

#### 7.2.2 Direct mTOR Kinase Inhibitor DES

The dysfunction of the endothelial barrier is one of the crucial causes of NA. Habib * et al*. [115] argued that a potential impairing mechanism of sirolimus on endothelial function is that it binds FK506 binding protein 12.6 kDa (FKBP12.6), which activates protein kinase C-α and disrupts the p120-VE cadherin interaction in the endothelium. Torin-2-eluting stents, which are a newer generation of stents that are adenosine triphosphate (ATP) selective and directly competitive with mTOR kinase, serve as a new generation of direct mTOR kinase inhibitor; they do not bind FKBP12.6, thus ensuring the reduction of injury to the endothelial barrier while resisting restenosis. In a rabbit stent implantation model, EES was shown to have negative effects on endothelial barrier function when compared to BMS, an effect that was mitigated when using a Torin-2 eluting stent [116]. Although Torin-2-eluting stents have not been adopted in clinical treatment, the refinement of molecular targeting of the mTOR complex can still be a promising strategy.

#### 7.2.3 Endothelial Cell Capture Stent

Recently, investigations on endothelial cell capture stents coated with monoclonal antibodies, such as cluster of differentiation (CD) 34, CD133 and CD146, have been carried out. In a prospective study, including 61 patients treated with dual-therapy endothelial progenitor cell-capturing SES, an anti-CD34 antibody-coated stent was shown by OCT to exhibit unique late neointimal regression, and it was first accompanied by good clinical results after 36 months, without late stent thrombosis [117]. Wawrzyńska *et al*. [118] reported that an anti-CD133 antibody stent accelerated re-endothelialisation and inhibited the proliferation of vascular smooth muscle cells, according to confocal images of endothelial cells and vascular smooth muscle cells, which can potentially avoid thrombosis and reduce restenosis. In comparison with BMS, the lumen area and stenosis area of the anti-CD146 antibody stent were reduced by 30%–60% [119]. To date, some achievements on endothelial cell capture stents of monoclonal antibodies CD34, CD133 and CD146 have been accomplished in animal models, but a horizontal comparison is lacking. Furthermore, the practical use of endothelial cell capture stents in clinical practice will need to be explored in the future.

### 7.3 Debulking Strategies

Calcification, one of the signs of advanced NA, can increase procedural difficulty in the following ways: (1) interference with lesion preparation and balloon dilation; (2) interference with the balloon and stent delivery; (3) restriction of stent expansion [120]. Calcium-ablation techniques and balloon-based therapies are the main therapies for calcified lesions.

#### 7.3.1 Calcium-Ablation Techniques

Rotational atherectomy (RA), orbital atherectomy system (OAS) and excimer laser coronary atherectomy (ELCA) are the available calcium-ablation techniques in clinical use, and they generally concentrate on modifying the plaque composition in the preparation of balloons and/or stent expansion.

The RA device is a diamond-tipped brass burr driven by the energy of the compressed gas. Sharma *et al*. [121] reported that in comparison with percutaneous transluminal coronary angioplasty, RA relatively inhibited intimal hyperplasia, lowering repeated stent use and decreasing the target vessel revascularisation rate. OAS reduces plaque burden with a mechanism aimed at minimising vessel wall trauma. A single-arm trial that enrolled 292 consecutive cases (374 lesions) who underwent PCI with OAS showed a 97% procedural success rate, major adverse cardiovascular events rates of 8% for myocardial infarction, 0.5% for cardiac death and 8% for target lesion revascularisation [122]. A meta-analysis involving 1872 patients showed that there were no significant differences between OAS and RA in relation to procedural, periprocedural, and thirty-day outcomes among patients with calcified CAD undergoing PCI [123]. The efficiency of ELCA has also been evaluated in small randomised studies of ISR patients. A 1-year follow-up evaluated the efficacy of ELCA + drug-coated balloon versus drug-coated balloon alone for 40 ISR patients, and showed that the former was more effective in preventing restenosis [124]. Although calcium ablation techniques are not considered a routine part of NA management, they can be utilised for the pretreatment of severely calcified lesions to ensure adequate balloon expansion.

#### 7.3.2 Balloon-Based Therapy

Balloon-based therapy refers to cutting balloons, scoring balloons, high-pressure balloons and intravascular lithotripsy.

The cutting balloon is a non-compliant balloon catheter equipped with 3 or 4 microblades. In the Cutting Balloon Global Randomised Trial, the primary endpoint, which was the 6-month binary restenosis, did not differ between cutting balloon and traditional balloon angioplasty (31% vs. 30%; *p* = 0.75), whereas the rate of perforation was higher with cutting balloon (0.8% vs. 0%; *p* = 0.03) [125]. A scoring balloon consists of a semi-compliant nylon balloon surrounded by three external nitinol spiral scoring wires. In an observational study of 299 patients undergoing IVUS-guided coronary DES implantation, AngioSculpt enhanced stent expansion in comparison with direct stenting and traditional balloon angioplasty with semi-compliant balloons [126]. The high-pressure balloon has a twin-layer structure with the capability of delivering high post-dilation pressures of >40 atm without bursting, making it the ‘last resort’ of dilation, especially suitable for stent under expansion in severe ISR with undilatable lesions or stents. A study involving 74 patients with severely calcified coronary artery lesions showed that preparation with a super high-pressure balloon versus a scoring balloon was associated with comparable stent expansion on intravascular imaging and a trend towards improved angiographic performance [127]. Inspired by extracorporeal shock-wave lithotripsy, a balloon catheter called intravascular lithotripsy was developed to disrupt coronary artery calcification. Recent studies have demonstrated the efficacy of intravascular lithotripsy in native AS [128, 129], but its role in NA remains to be clarified.

## 8. Conclusions

NA remains a major issue to be solved after stenting. The potential mechanisms of NA mainly point to incomplete endothelialisation, haemodynamic changes and stent-induced inflammatory processes. Great efforts have been made to improve various treatments, with the aim of trying to control the development of NA. While there has been progress in clinical improvement in NA incidences with various technical improvements, an incomplete understanding of the underlying pathologic processes has still hampered its prevention. More basic and clinical research is required to lay the foundation for NA exploration in the future.

## Abbreviations

NA, neoatherosclerosis; PCI, percutaneous coronary intervention; ISR, in-stent restenosis; ISNA, in-stent neoatherosclerosis; DES, drug-eluting stents; BMS, bare metal stents; IVUS, intravascular ultrasound; OCT, optical coherence tomography; AS, atherosclerosis; TCFA, thin-cap fibrous atherosclerosis; NIRS, near infrared spectroscopy; NIRF, near infrared fluorescence; BVS, bioresorbable vascular scaffolds; SES, sirolimus-eluting stent; PES, paclitaxel-eluting stent; EES, everolimus-eluting stent; VLST, very late stent thrombosis; CD, cluster of differentiation; mTOR, mammalian target of rapamycin; RA, rotational atherectomy; OAS, orbital atherectomy system; ELCA, excimer laser coronary atherectomy.
